# Supplementary material for: Substantial oxygen consumption by aerobic nitrite oxidation in oceanic oxygen minimum zones
Source: Nat Commun. 2021 Dec 2;12:7043. doi: 10.1038/s41467-021-27381-7 (PMC8639706; doi:10.1038/s41467-021-27381-7)
Supplement: Supplementary file 1 — Supplementary Information [file 41467_2021_27381_MOESM1_ESM.pdf]

Supplementary materials for this manuscript include the following:

Supplementary Notes 1-2

Supplementary Figures 1-5

Supplementary Tables 1-2

Supplementary Methods

Supplementary Note 1

Two approaches have been used to examine the oxygen sensitivity of oxygen consumption rates (OCR) in previous work. Tiano et al.[1] used changes in the slope of the DO-time relationship to calculate oxygen affinities at DO concentrations <500 nM, reporting two values from the ETNP. Garcia-Robledo et al.[2] used this approach for a single incubation, while also reporting values based on multiple incubations conducted across a range of DO concentrations. Although these two approaches display the same type of pattern—OCR decreases as DO decreases—they differ in their details, because they focus on distinct properties of microbial communities. As Tiano et al.[1] note, "there may be two or more populations with very different K_m values in the same water layer."

The time course or depletion curve approach examines DO affinity only at low DO concentrations: Tiano et al.[1], for instance, limited their analysis in the ETNP to depletion curves at DO concentrations <500 nM. These measurements likely reflect only a subset of the community possessing enzymes with high affinity for DO—including some nitrite oxidizers[3,4,5]. Using the approach of Tiano et al.[1], we calculated high DO affinities of 53-127 nM DO within incubation bottles showing nonlinearity in DO consumption. These affinities overlap with those of Tiano et al.[1] (18-136 nM).

The overall pattern, based on multiple bottles and a wider range of DO values, reflects mixed assemblages of microorganisms that use DO to oxidize a variety of substrates. These substrates can include nitrite, but especially different forms of organic matter, and possibly reduced sulfur compounds and methane[6]. Within these different functional groups, different lineages may possess mutliple enzymes of varying oxygen affinities[3,4,5]. Finally, variations in organic matter composition may also affect which microbial groups are actively respiring organic matter, as well as the energetic yield under different DO concentrations. As Tiano et al.[1] state: "A simple Michaelis–Menten…model is thus an oversimplification." This applies to

organisms respiring organic carbon, but also applies to nitrite oxidizers. As Bristow et al.[5] show, even within nitrite oxidizers alone, the "oxygen response likely represents the response of a mixed community potentially carrying a variety of terminal oxidases with different K_m values."

Kinetic parameters calculated from incubations conducted at different DO levels are therefore uniformly larger in magnitude than those calculated from oxygen depletion curves. For example, Garcia-Robledo et al.[2] calculated an affinity of 38 nM DO based on one depletion curve, but the overall K_m value for all incubations considered together was over an order of magnitude higher. Variability in their data also shows that OCR increases up to ~2000 nM DO, with lower rates then measured at >5000 nM, resulting in a lower r^2 value of 0.342. Tiano et al.[1] calculated high affinities (small K_m) from depletion curves in some bottles, but their data showed that maximum rates occurred at higher DO concentrations: rates were maximal at 1500-1700 nM DO at 30 m depth at station M1, 1000-1300 nM at 40 m, and 1400-1450 nM at 300 m.

Our experimental results are generally consistent with these two earlier studies, with some differences. First, like both studies, we do calculate high affinities when using the approach of Tiano et al.[1] applied to DO time courses in individual bottles—yet across multiple incubations conducted at different DO levels, OCR increases into the µM DO range (Table 1). For the reasons discussed above, these distinct approaches give distinct answers.

Second, the general pattern of increasing OCR with increasing DO is consistent with earlier observations in that OCR increases up to the µM DO range—i.e., this does not occur at substantially higher or lower DO concentrations in our experiments. We also note that scatter in the Garcia-Robeldo et al.[2] data may affect their calculations, while Tiano et al.[1] only used a single approach to calculate K_m, and the overall range of DO concentrations that they used was more limited. However, it is the case that OCR levels off at slightly higher DO concentrations in some of our experiments. This results in overall K_m values mostly in the 1-2 µM range, with several higher than this (Table 1). However, two of the higher values occurred in OMZ edge samples, where significantly more organic matter is present (Table 1). In addition, organic matter composition also varies with depth—as well as from location to location—which may affect OCR. The station 3 SNM experiment K_m estimate is particularly high, but µM levels of DO are never likely to occur *in situ* in the SCM or SNM, and were included for purposes of comparison. These values can influence the calculation of kinetic parameters when compared with incubations conducted at lower DO (100s of nM). However, we are focused on OCR and

nitrite oxidation rates at these lower DO concentrations, where rates scale with DO. Finally, Kalvelage et al.[3] consistently showed that OCR continued to increase with increasing DO, up to the highest DO concentrations that they used (10s of μM). This suggests further complexity and variability in the overall response of OCR to DO.

In sum, our low-level/high affinity K_m values are highly consistent with earlier work[1]. Our overall kinetic parameter results are slightly higher than some previous studies[1,2], while considerably lower than other work[4]. They are furthermore consistent with model predictions[7,8].

Supplementary Note 2

In addition to calculating kinetic parameters for OCR data, we also calculated K_m and v_{max} values for nitrite oxidation rates across relevant incubation bottles (although, again, this is an oversimplification for nitrite oxidation alone, as these are mixed microbial assemblages[5]). High DO affinities (low K_m values) were observed for nitrite oxidation in all experiments, but particularly in the SCM (Stations 2 and 3) and SNM (Stations 3 and 3.5). K_m values ranged from 34.1-185 nM DO in the SCM and SNM, whereas OMZ edge values ranged from 346-1876 nM DO. Low K_m values (higher affinities) for DO for nitrite oxidation in the SCM and SNM are consistent with low-level K_m values for OCR (calculated from DO consumption over time)—lending additional support to the idea that nitrite oxidation is important for DO consumption at concentrations <200 nM DO.

K_m values for nitrite oxidation were also similar to, and in some cases lower than, previously reported values that fall in the 100s of nM DO[5,9]. Our results are also consistent with the idea that different assemblages present at different depths can vary in their affinity for DO[5,9]: in fact, Bristow et al.[5] argue for a two-component fit to their data, although the high affinity component was poorly constrained. Finally, we also observed limited evidence for DO inhibition of nitrite oxidation[9,10] at DO concentrations >5 μM in the SNM (Fig. 4D and F). This underlines the importance of using a range of relevant of DO concentrations, as measurements predominantly in the micromolar range may capture fundamentally different behavior than measurements in the sub-micromolar range, and the high DO affinity of nitrite oxidation cannot be as accurately resolved.

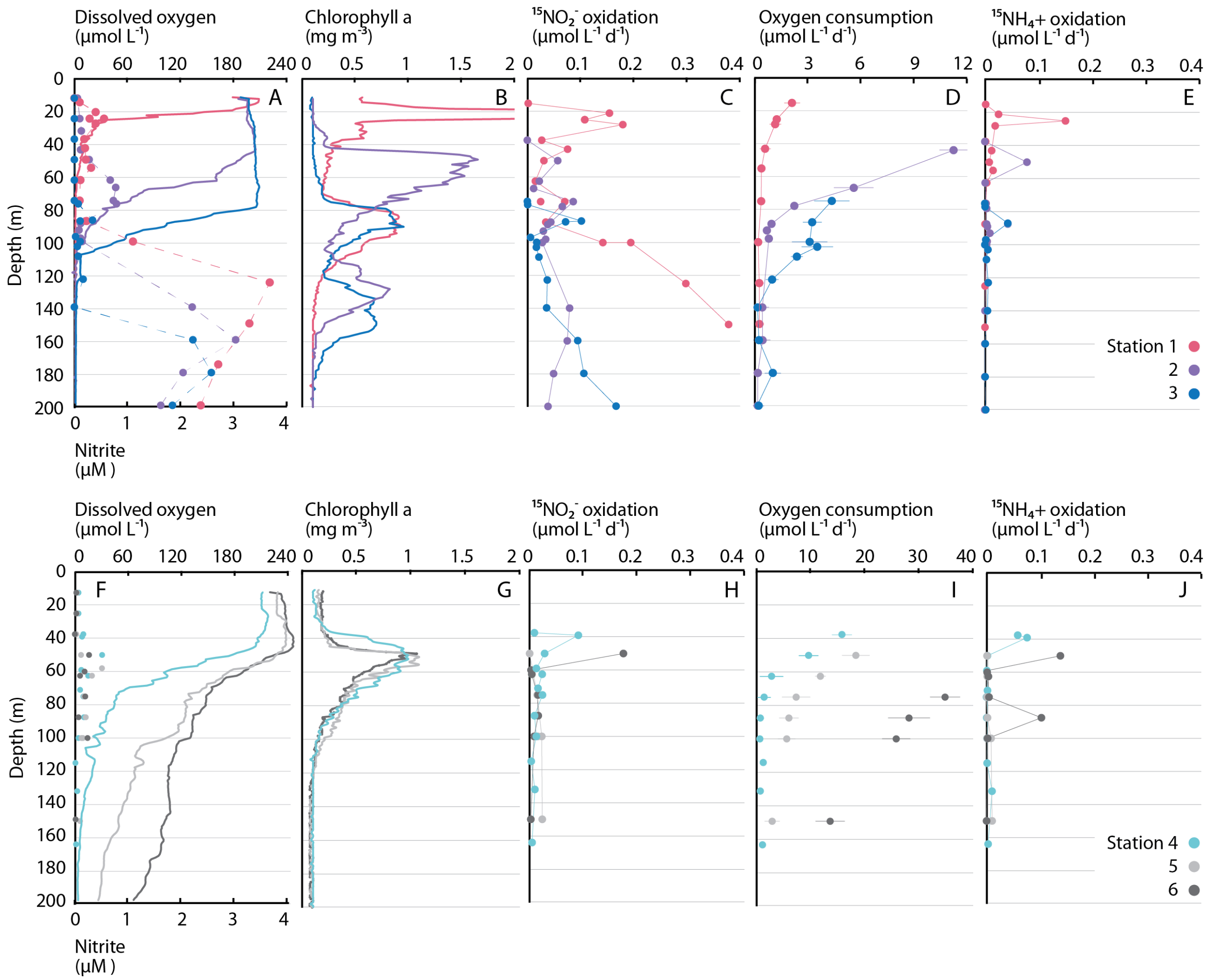

Supplementary Figure 1: Depth profiles of (A and F) dissolved oxygen (solid lines) and nitrite (data points); (B and G) chlorophyll a; (C and H) nitrite oxidation rates; (D and I) OCR (data presented as mean values of five independent replicates ±1 SD); and (E and J) ammonia oxidation rates. Stations are denoted by different colors, with Stations 1-3 shown in panels (A-E) and Stations 4-6 in panels (F-J). Panels A-D are identical to Figure 2 and included together here for comparison. Maximum chlorophyll values at Station 1 plot off-axis. For Stations 5 and 6 in panel F, dissolved oxygen does not drop to 20 µM over the plotted depth range, as this occurs deeper within the water column.

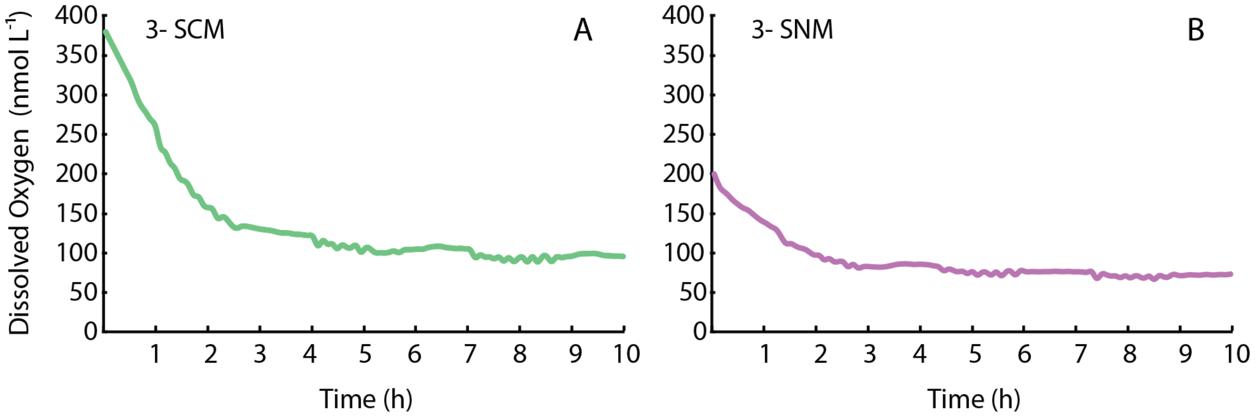

Supplementary Figure 2: Examples of DO time courses showing declining OCR with decreasing DO (see main text) during oxygen manipulation experiments. Five minute averages of dissolved oxygen concentrations measured using trace oxygen sensor spots (FireSting, Pyroscience) are shown for the first ten hours of incubations conducted in the (A) secondary chlorophyll maximum (SCM) and (B) secondary nitrite maximum (SNM) at Station 3.

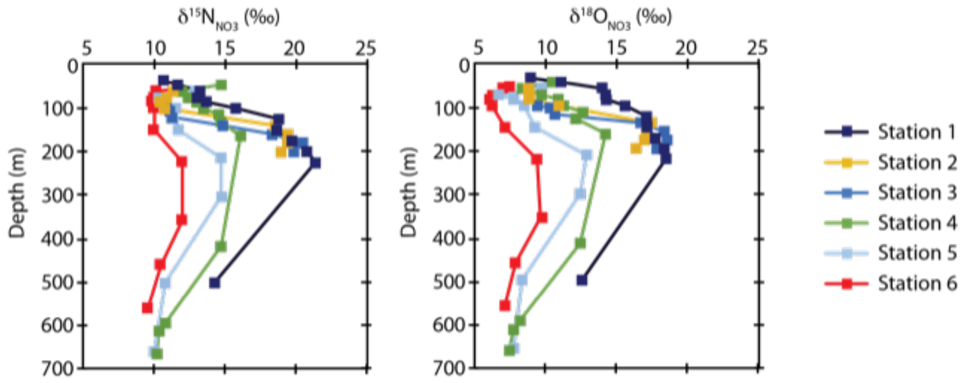

Supplementary Figure 3: Depth profiles of the $\delta^{15}N$ and $\delta^{18}O$ stable isotopic composition of dissolved nitrate. Colors denote different sampling stations.

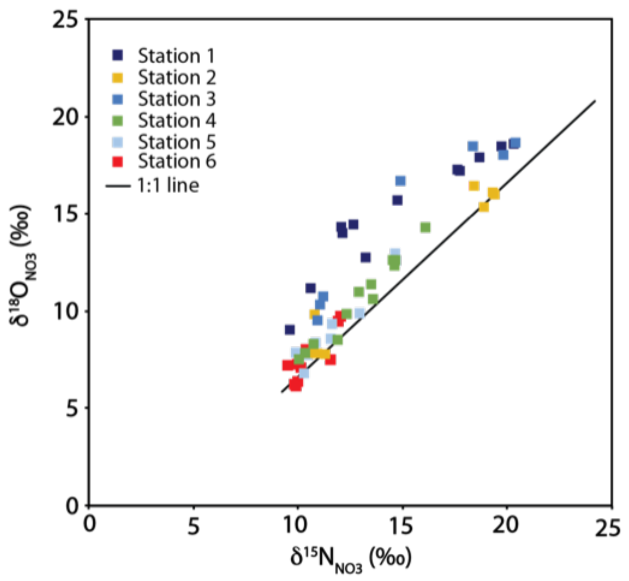

Supplementary Figure 4: Dual isotope plot of the δ^{15}N and δ^{18}O stable isotopic composition of dissolved nitrate. Colors denote different sampling stations and black line denotes a 1:1 relationship.

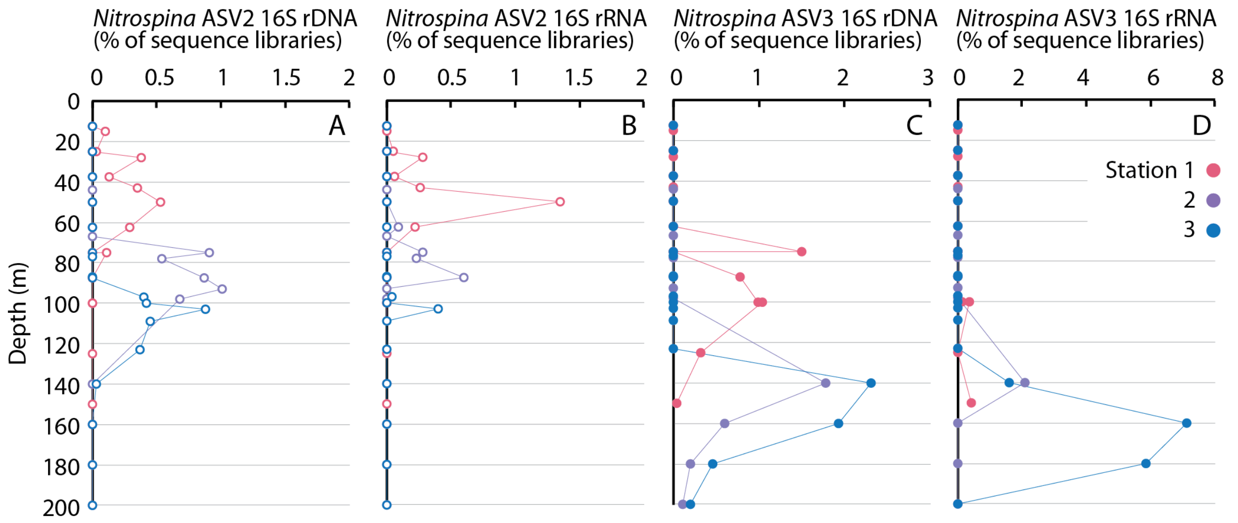

Supplementary Figure 5: Depth profiles of (A and B) *Nitrospina* amplicon sequence variant (ASV) 2 (open symbols) and (C and D) *Nitrospina* ASV3 (filled symbols) as a percentage of (A and C)16S rDNA sequence libraries and (B and D) 16S rRNA sequence libraries. ASV2 is active in the upper water column, whereas ASV3 is dominant in the OMZ. Colors denote different sampling stations.

Supplementary Table 1: Relative abundance of all *Nitrospina* reads and relevant *Nitrospina* functional genes within metagenomes (expressed per million reads) collected on the edge of the oxygen minimum zone (OMZ), in the secondary chlorophyll maximum (SCM), and in the secondary nitrite maximum (SNM) at Stations 1-3.

**Sta.**	**Depth**	**Type**	**Initial DO (μM)**	***Nitrospina* (all reads)**	***Nitrospina* Nitrite oxido-reductase (*nxr*)**	***Nitrospina* Chlorite dismutase (*Cld*)**	***Nitrospina* Nitrate reductase (*nar*)**	***Nitrospina* Formate dehydrogenase (*Fdh*)**
1	25	OMZ edge	19.9	1084	2	1	0	0
1	87.5	SCM	0.46	31920	65	16	23	3
1	100	SNM	0.30	21196	37	7	30	7
2	89	OMZ edge	15.3	22442	38	14	0	0
2	130	SCM	0.78	33290	57	11	22	4
2	160	SNM	0.60	21725	37	6	36	7
3	123	OMZ edge	13.0	18647	34	11	1	1
3	140	SCM	0.74	34227	55	12	23	4
3	180	SNM	0.45	17617	30	6	35	5

Initial dissolved oxygen (DO) concentrations were measured in unmanipulated incubation bottles using oxygen sensor spots (Fibox, Loligo) immediately following sample collection.

Supplementary Table 2: Conductivity-temperature-depth (CTD) dissolved oxygen (DO) sensor values for each depth sampled for joint nitrite oxidation and OCR measurements.

Station	Depth	CTD DO (µM)
1	15	32.40
1	25	21.90
1	28	23.26
1	37.5	7.80
1	43	6.20
1	50	4.60
1	62.5	1.40
1	75	1.30
1	87.5	b.d.
1	100	b.d.
1	125	b.d.
1	150	b.d.
2	37.5	207.70
2	50	181.20
2	62.5	119.96
2	67	99.50
2	75	41.70
2	78	40.50
2	87.5	12.90
2	89	14.50
2	93	9.20
2	98	7.00
2	100	3.93
2	140	b.d.
2	160	b.d.
2	180	b.d.
2	200	b.d.
3	75	215.05
3	87.5	214.57
3	100	146.72
3	103	55.90
3	109	39.10
3	123	11.90
3	140	b.d.
3	160	b.d.

3	180	b.d.
3	200	b.d.
4	37.5	221.42
4	39	217.55
4	50	177.70
4	59	136.07
4	62.5	108.35
4	71	86.13
4	75	59.30
4	87.5	40.20
4	100	28.36
4	115	20.23
4	132	14.72
4	164	5.60
4	415	1.33
4	595	1.43
5	50	241.12
5	62.5	188.33
5	75	153.71
5	87.5	163.08
5	100	115.72
5	150	58.50
5	217	22.20
5	304	9.62
5	500	5.44
5	660	3.84
6	50	226.61
6	60	196.80
6	62.5	195.50
6	75	148.56
6	87.5	136.91
6	100	118.90
6	150	106.91
6	228	51.31
6	360	20.04
6	460	9.34
6	560	6.50

b.d. = Below the detection limit (1 μM) of the CTD DO sensor.

Supplementary Methods

Nutrient Analyses

Samples were analyzed at sea for NH_4^+ using the fluorescent method of Holmes et al. (11), and for NO_2^- using the sulfanilamide method, both on a Trilogy Laboratory Fluorometer (Turner Designs; San Jose, CA, USA) equipped with the NH_4^+ or NO_2^- module. Standards ranged from 0 to 186 nM for NH_4^+ and 0 to 10,000 nM for NO_2^-, and all standard curve r^2 values were >0.99. Samples for combined NO_3^-+NO_2^- and for PO_4^{3-} were frozen aboard the ship and subsequently analyzed at UCSB Marine Science Institute Analytical Lab. Combined NO_3^-+NO_2^- were measured using EPA Method 353.2 (ref. 12), and PO_4^{3-} using EPA Method 365.1 (ref. 13), on a QuikChem 8500 Series 2 Flow Injection Analyzer (Lachat Instruments, Zellweger Analytics; Concord, ON, CA). A mid-range check standard took place every 20 samples to verify the accuracy of the measurements. Detection limits were 0.20 μM for NO_3^- + NO_2^- and 0.10 μM for PO_4^{3-} and standard curve r^2=0.996.

RNA and DNA Extraction

Water samples were collected for RNA and DNA extraction using sampling bottles deployed on the CTD rosette. At each depth, parallel sets of 2L samples were filtered through 0.22 μm filters (Millipore, Darmstadt, Germany) using a peristaltic pump. RNA filter samples were filtered within 15 minutes of collection, submerged in RNA*later*® (Ambion™, AM7021) in pre-prepped Lysing Matrix E tubes (MP Bio, Eschwege, Germany), and frozen at -80°C until extraction. DNA filter samples were submerged in in Sucrose-Tris-EDTA (STE) buffer in pre-prepped Lysis Matrix E tubes and frozen at -80°C until extraction.

RNA was extracted using the *mir*Vana RNA Isolation kit (Ambion™, AM1560) following a protocol modified from Huber and Fortunato (protocols.iodx.doi.org/10.17504/protocols.io.iuxcexn). In brief, extraction space was prepared by wiping the bench top, fume hood, pipettes, and racks with 10% bleach, 70% ethanol, and RNAzap wipes. Filters were transferred to 5mL tubes and vortexed with lysis buffer for 7-10 minutes at setting 6; each sample then received 1.25 volumes of room temperature 100% ethanol, was bound to the filter cartridge, and purified and eluted following the manufacturer's instructions. cDNA was generated from extracted RNA using the Invitrogen SuperScript III Reverse Transcriptase kit (Life Technologies Corporation, Carlsbad, CA, USA) following the

manufacturer's instructions. Briefly, 5 µl of RNA extract, 1 µl each of OligodT and 1 µl 10mM dNTPs were incubated at 65°C for 5min, and then placed on ice for 1 min; samples were then incubated with 10 µl of cDNA synthesis mix for 25°C for 10 min, then 55 °C for 50 min, and the RT reaction was terminated at 85 °C for 5min. Remaining RNA was removed through incubation with RNase H (Invitrogen) at 37 °C for 20 min.

DNA was extracted from filters following Beman et al.[14]. Briefly, 100µL 10% sodium dodecyl sulfate (SDS) was added to tubes containing STE buffer and filters; samples were bead-beat for 2 minutes (BioSpec Products, Inc., Bartlesville, OK, USA) and then incubated for 3 minutes on a dry heat block at 99°C. Following transfer of sample solutions to 1.5 mL LoBind Microcentrifuge tubes (Eppendorf, Hauppauge, NY, USA), 50µL proteinase K (20mg mL^{-1}; Qiagen, Inc., Valencia, CA, USA) was added, and tubes were incubated at 55°C for 3 hours. Lysates were purified using the Qiagen DNeasy Blood and Tissue Kit (Qiagen, Inc., Valencia, CA, USA).

16S rRNA Sequencing and Sequence Processing

cDNA and DNA samples were diluted to a common concentration (1 ng/µL) and sent to Argonne National Laboratory (Lemont, IL, USA) for 16S rRNA amplicon sequencing on an Illumina MiSeq (Illumina, San Diego, CA, USA) according to Earth Microbiome protocols. We used the universal primers 515F-Y (5'-GTGYCAGCMGCCGCGGTAA) and 926R (5'-CCGYCAATTYMTTTRAGTTT), which are effective for analysis of marine microbial communities[15]. In total, 73 DNA samples and 73 corresponding RNA samples were sequenced.

ASVs were generated from 16S rDNA and rRNA sequence data using the Divisive Amplicon Denoising Algorithm (DADA2; ref. 16) as implemented in QIIME 2 (ref. 17), and then used for subsequent analyses. After import and demultiplexing, read quality was visualized using the 'qiime tools view' command. Reads were then processed using the 'qiime dada2 denoise-paired' command, with 13 bp trimmed from both the forward and reverse reads, truncation of reverse to 169 bp (due to the well-known decline in sequence quality observed for MiSeq reverse reads), and training of the denoising algorithm on 1 million reads. Classification of ASVs was conducted in mothur[18] using the SILVA (version 128) database.

Metagenome Sequencing and Analysis

Following extraction, DNA samples were sent for metagenome sequencing in the Vincent J. Coates Genome Sequencing Laboratory (GSL) at the University of California, Berkeley (https://genomics.qb3.berkeley.edu/), which is supported by NIH S10 OD018174 Instrumentation Grant. For each sample, 250 ng of genomic DNA was sheared and libraries were prepared using the KAPA HyperPrep Kit (Kapa Biosystems, Wilmington, MA, USA). 16 samples (including the nine reported here) were pooled into a single lane and sequenced via 150-cycle paired-end sequencing on the Illumina HiSeq 4000 platform (Illumina, Inc., San Diego, CA, USA).

Data were demultiplexed by the GSL and reads were filtered and trimmed using BBDuk (https://jgi.doe.gov/data-and-tools/bbtools/bb-tools-user-guide/bbduk-guide/) with the following parameters: maq=8, maxns=1, minlen=40, minlenfraction=0.6, k=23, hdist=1, trimq=12, qtrim=rl. Forward and reverse reads were then merged using PANDASeq (https://github.com/neufeld/pandaseq; ref. 19) with default parameters. Merged reads were subsequently annotated in DIAMOND (http://diamondsearch.org/; ref. 20) using the NCBI NR database (accessed February 11th, 2020) with maximum number of target sequences = 1 and bit-score > 40. We used grep to search for genes and organismal groups of interest listed in the text.

Organic Matter Analysis

Seawater for [TOC] analysis was collected directly into combusted 40-mL borosilicate vials and sealed using acid-washed vial caps with septa. Samples were immediately acidified to pH 2 using trace metal grade 12N HCl (Fisher Scientific), and stored at room temperature in the dark until analyzed via high-temperature combustion on a Shimadzu 500 V-CSN/TNM-1 (Shimadzu Corp, Kyoto, Japan). The Shimadzu modified from the manufacturer's design, and the combustion oven contained a quartz column filled with platinum (Pt) catalyst beads. Combustion columns were pre-conditioned on 40 - 100 injections of filtered (0.2 µm) seawater until the baseline of measured carbon was stable. A magnesium perchlorate water-trap was placed prior to the halogen trap and changed daily. CO_2-free carrier gas was used to pre-condition the column, and ultra-high purity grade O_2 gas was delivered to the instrument as the carrier gas during sample analysis.

Each acidified TOC sample was sparged for two minutes and measured following high-temperature combustion at 680˚ C. During analysis, five 100 µL injections were made from a single sample reservoir, and samples were reanalyzed when the %CV of the best 3 injections was >5%. TOC measurements were calibrated using an 8-point calibration curve between 10 and 100 µM C of potassium phthalate in Milli-Q water. Milli-Q water and a reference water sample were analyzed every 10 samples. Reference standards were obtained from NSF-supported deep Florida Strait (Batch 6FS – 2006; Dennis Hansell, RSMAS, University of Miami). The expected concentration range for Batch reference materials is provided at http://www.rsmas.miami.edu/groups/biogeochem/.

For organic matter composition, 1L seawater was solid phase extracted[21] and analyzed using liquid chromatography coupled with high-resolution tandem mass spectrometry according to Petras et al.[22]. After removal of contaminants, the intensities of 6759 molecular features were relativized and angular transformed, and Bray-Curtis dissimilarity was used in principal coordinates analysis.

Supplementary References

1. L. Tiano, Laura, *et al.*, Oxygen distribution and aerobic respiration in the north and south eastern tropical Pacific oxygen minimum zones. *Deep Sea Re.s Part I: Oceanogr. Res. Papers* **94**: 173-183 (2014).
2. E. Garcia-Robledo, *et al.*, Determination of respiration rates in water with sub-micromolar oxygen concentrations. *Front. Mar. Sci.* 3: 244 (2016).
3. E. Garcia-Robledo, *et al.*, Cryptic oxygen cycling in anoxic marine zones. *Proc. Natl. Acad. Sci. U. S. A.* **114**, 8319–8324 (2017).
4. T. Kalvelage, *et al.*, Aerobic Microbial Respiration In Oceanic Oxygen Minimum Zones. *PLoS One* **10**, e0133526 (2015).
5. L. A. Bristow, *et al.*, Ammonium and nitrite oxidation at nanomolar oxygen concentrations in oxygen minimum zone waters. *Proc. Natl. Acad. Sci. U. S. A.* **113**, 10601–10606 (2016).
6. M. Holtappels, *et al.*, Aquatic respiration rate measurements at low oxygen concentrations. *PLoS One* **9** (2014).

7. J. L. Penn, T. Weber, B. X. Chang, C. Deutsch, Microbial ecosystem dynamics drive fluctuating nitrogen loss in marine anoxic zones. *Proc. Natl. Acad. Sci. U. S. A.* **116**, 7220–7225 (2019).

8. C. Deutsch, H. Brix, T. Ito, H. Frenzel, L. A. Thompson, Climate-forced variability of ocean hypoxia. *Science* **333**, 336–339 (2011).

9. X. Sun, *et al.*, Microbial niche differentiation explains nitrite oxidation in marine oxygen minimum zones. *ISME J.* (2021).

10. X. Sun, Q. Ji, A. Jayakumar, and B.B Ward, Dependence of nitrite oxidation on nitrite and oxygen in low‐oxygen seawater. *Geophysical Res. Lett.* **44**, 7883-7891 (2017).

11. R. M. Holmes, A. Aminot, R. Kérouel, B. A. Hooker, B. J. Peterson, A simple and precise method for measuring ammonium in marine and freshwater ecosystems. *Can. J. Fish. Aquat. Sci.* **56**, 1801–1808 (1999).

12. D. Diamond, Determination of nitrate in brackish or seawater by flow injection analysis. *QuikChem Method*, 31-107-04-1A. (1999).

13. A. Huberty, D. Diamond, Determination of phosphorus by flow injection analysis colorimetry. *QuikChem Method*, 31–115 (1998).

14. J. M. Beman, B. N. Popp, S. E. Alford, Quantification of ammonia oxidation rates and ammonia-oxidizing archaea and bacteria at high resolution in the Gulf of California and eastern tropical North Pacific Ocean. *Limnol. Oceanogr.* **57**, 711–726 (2012).

15. A. E. Parada, D. M. Needham, J. A. Fuhrman, Every base matters: Assessing small subunit rRNA primers for marine microbiomes with mock communities, time series and global field samples. *Environ. Microbiol.* **18**, 1403–1414 (2016).

16. B. J. Callahan, P. J. McMurdie, M. J. Rosen, A. W. Han, A. J. A. Johnson, S. P. Holmes, DADA2: high-resolution sample inference from Illumina amplicon data. *Nature Meth.* **13**, 581-583 (2016).

17. E. Bolyen, *et al.*, Reproducible, interactive, scalable and extensible microbiome data science using QIIME 2. *Nat. Biotechnol.* **37**, 852–857 (2019).

18. P. D. Schloss, *et al.*, Introducing mothur: Open-source, platform-independent, community-supported software for describing and comparing microbial communities. *Appl. Environ. Microbiol.* **75**, 7537–7541 (2009).

19. A. P. Masella, A. K. Bartram, J. M. Truszkowski, D. G. Brown, J. D. Neufeld, PANDAseq: Paired-end assembler for illumina sequences. *BMC Bioinformatics* **13**, 1–7 (2012).
20. B. Buchfink, C. Xie, D. H. Huson, Fast and sensitive protein alignment using DIAMOND. *Nat. Methods* **12**, 59–60 (2014).
21. T. Dittmar, et al. A simple and efficient method for the solid‐phase extraction of dissolved organic matter (SPE‐DOM) from seawater. *Limnology and Oceanography: Methods* **6**, 230-235 (2008).
22. D. Petras, et al. High-resolution liquid chromatography tandem mass spectrometry enables large scale molecular characterization of dissolved organic matter. *Front. Mar. Sci.* **4**, 405 (2017).
