# Supplementary material for: Substantial oxygen consumption by aerobic nitrite oxidation in oceanic oxygen minimum zones
Source: Nat Commun. 2021 Dec 2;12:7043. doi: 10.1038/s41467-021-27381-7 (PMC8639706; doi:10.1038/s41467-021-27381-7)
Supplement: Supplementary file 3 — Reporting Summary [file 41467_2021_27381_MOESM3_ESM.pdf]

nature research

Corresponding author(s): Beman

Last updated by author(s): Oct 19, 2021

Reporting Summary

Nature Research wishes to improve the reproducibility of the work that we publish. This form provides structure for consistency and transparency in reporting. For further information on Nature Research policies, see our Editorial Policies and the Editorial Policy Checklist.

Statistics

For all statistical analyses, confirm that the following items are present in the figure legend, table legend, main text, or Methods section.

n/a	Confirmed	
☐	☒	The exact sample size (*n*) for each experimental group/condition, given as a discrete number and unit of measurement
☐	☒	A statement on whether measurements were taken from distinct samples or whether the same sample was measured repeatedly
☐	☒	The statistical test(s) used AND whether they are one- or two-sided<br>*Only common tests should be described solely by name; describe more complex techniques in the Methods section.*
☐	☒	A description of all covariates tested
☒	☐	A description of any assumptions or corrections, such as tests of normality and adjustment for multiple comparisons
☐	☒	A full description of the statistical parameters including central tendency (e.g. means) or other basic estimates (e.g. regression coefficient) AND variation (e.g. standard deviation) or associated estimates of uncertainty (e.g. confidence intervals)
☐	☒	For null hypothesis testing, the test statistic (e.g. *F*, *t*, *r*) with confidence intervals, effect sizes, degrees of freedom and *P* value noted<br>*Give P values as exact values whenever suitable.*
☒	☐	For Bayesian analysis, information on the choice of priors and Markov chain Monte Carlo settings
☒	☐	For hierarchical and complex designs, identification of the appropriate level for tests and full reporting of outcomes
☒	☐	Estimates of effect sizes (e.g. Cohen's *d*, Pearson's *r*), indicating how they were calculated

Our web collection on statistics for biologists contains articles on many of the points above.

Software and code

Policy information about availability of computer code

Data collection	None used
Data analysis	RStudio (v1.0.136), R package drc (v3.0-1), Divisive Amplicon Denoising Algorithm (DADA2; v1.12) implemented in QIIME 2 (v2019.7.0), mothur (v1.42.2), BBDuk (v38.23), PANDASeq (v2.11), DIAMOND (v0.9.30).

For manuscripts utilizing custom algorithms or software that are central to the research but not yet described in published literature, software must be made available to editors and reviewers. We strongly encourage code deposition in a community repository (e.g. GitHub). See the Nature Research guidelines for submitting code & software for further information.

Data

Policy information about availability of data

All manuscripts must include a data availability statement. This statement should provide the following information, where applicable:

- Accession codes, unique identifiers, or web links for publicly available datasets
- A list of figures that have associated raw data
- A description of any restrictions on data availability

The CTD data generated in this study have been deposited in the Rolling Deck to Repository, with 2017 data available under accession number OC1704A [https://www.rvdata.us/search/cruise/OC1704A], and 2018 data available under accession number OC1806A [https://www.rvdata.us/search/cruise/OC1806A]. Nutrient and biogeochemical rate data are available through the Biological and Chemical Oceanography Data Management Office under project number 863208 [https://www.bco-dmo.org/project/863208]. Sequence data are available in the Sequence Read Archive, with 16S data available under BioProject PRJNA192803 [https://www.ncbi.nlm.nih.gov/bioproject/PRJNA192803], and metagenome data available under BioProject PRJNA634212 [https://www.ncbi.nlm.nih.gov/bioproject/PRJNA634212].

Field-specific reporting

Please select the one below that is the best fit for your research. If you are not sure, read the appropriate sections before making your selection.

☐ Life sciences ☐ Behavioural & social sciences ☒ Ecological, evolutionary & environmental sciences

For a reference copy of the document with all sections, see nature.com/documents/nr-reporting-summary-flat.pdf

Ecological, evolutionary & environmental sciences study design

All studies must disclose on these points even when the disclosure is negative.

Study description	Nitrite oxidation rate measurements (using 15NO2-), OCR measurements (using optical sensor spots), and ammonia oxidation rate measurements (using 15NH4+) were made along depth profiles at six stations in the ETNP, including three OMZ stations and three AMZ stations. Other measurements included water column properties, stable isotopic composition of dissolved nitrate, and 16S rRNA and metagenome sequencing.
Research sample	At each station, we sampled in the upper 100 m to capture the primary nitrite maximum and an expected peak in nitrite oxidation rates at the base of the euphotic zone (EZ). We then sampled across a range of DO levels and nitrite levels to quantify rate variations in response to vertical gradients in DO. Samples were collected at 200, 100, 50, 20, 10, 5, and 1 µM [DO] at all stations.
Sampling strategy	OCR measurements were made with five replicates, providing sufficient replication to accurately measure rates. Ammonia/nitrite oxidation rate measurements, isotopic measurements, and 16S/metagenome sequencing represent single measurements owing to the expense of the analyses and their general reproducibility.
Data collection	Data were recorded in field and lab notebooks while at sea or in the lab by JMB, JMW, and EPC, with assistance from the other authors, and then immediately transferred to spreadsheets.
Timing and spatial scale	Samples were collected in April 2017 and June 2018 (in order to capture any annual variation in conditions) aboard the R/V Oceanus. The sampling region ranged from 16 to 27.4 N and 106.5 to 117.5 W.
Data exclusions	For water column profiles, OCR rate values calculated at 10-14 hour and 20-24 hour measurement time points were highly correlated with each other (r2= 0.968-0.995; slopes=1.06-1.19; all P<0.0001 across different stations), indicating that OCR did not accelerate or decrease substantially over the course of the incubations. The only exceptions were three sampling depths from station 3 (77, 87, 97 m) showing nonlinearity; following the suggestions of the reviewers, we instead use data from duplicate OCR measurements conducted at similar depths (75, 88, 100 m) during the previous 24 hour period (when nitrite oxidation rates were not measured in tandem).
Reproducibility	Oxygen manipulation experiments were conducted in 500 mL serum bottles with attached FireSting sensor spots. Experiments were repeated successfully 7 times. For each experiment, a total of 24 bottles were filled with water collected at a specific depth, sealed, and then bubbled with ultrapure He gas while DO was monitored. 8 bottles had tracer-level 15NO2- additions, 8 bottles had tracer-level 15NH4+ additions, and 8 were unlabeled. In each set of 8 bottles, we established initial DO values typically ranging from 10s to 1000s of nM. OCR was measured in all bottles based on starting and ending DO values, and samples for nitrite oxidation were collected from 15NO2- labeled bottles at the end of the experiments. For all experiments, dedicated bottles were used for the different 15N labels.
Randomization	In all cases, different depths/water samples were collected randomly into differently numbered sample bottles. For experiments, bottles were allocated randomly into treatment groups. Bottle numbers were recorded while sampling, and only bottle numbers (not sample numbers) were noted during data collection phase. After analysis, bottle numbers and samples were matched.
Blinding	All measurements in the field and lab were made on numbered samples, such that all analyses were blinded.

Did the study involve field work? ☒ Yes ☐ No

Field work, collection and transport

Field conditions	Air and water temperatures increased from north to south given the significant differences in latitude. Conditions were calm in 2017; however, in 2018, a Category 4 hurricane disrupted sampling.
Location	Station locations are provided in Figure 1, and details of sample depths are provided in the Supplement.
Access & import/export	Samples collected in territorial waters of Mexico were collected under Instituto Nacional de Estadística y Geografía (INEGI) permits EG0062017 and EG0032018, and Permiso de Pesca de Fomento permits PPFE/DGOPA-016/17 and PPFE/DGOPA-027/18.
Disturbance	None

Reporting for specific materials, systems and methods

We require information from authors about some types of materials, experimental systems and methods used in many studies. Here, indicate whether each material, system or method listed is relevant to your study. If you are not sure if a list item applies to your research, read the appropriate section before selecting a response.

Materials & experimental systems

n/a	Involved in the study
☒	☐ Antibodies
☒	☐ Eukaryotic cell lines
☒	☐ Palaeontology and archaeology
☒	☐ Animals and other organisms
☒	☐ Human research participants
☒	☐ Clinical data
☒	☐ Dual use research of concern

Methods

n/a	Involved in the study
☒	☐ ChIP-seq
☒	☐ Flow cytometry
☒	☐ MRI-based neuroimaging
